# Object and Spatial Context Representations in Visual Short-Term Memory

**DOI:** 10.1523/ENEURO.0076-21.2021

**Published:** 2021-04-15

**Authors:** Aedan Y. Li

**Affiliations:** Department of Psychology, University of Toronto, Toronto, ON M5S, Canada

## Abstract

**Highlighted Research Paper:** The Role of Location-Context Binding in Nonspatial Visual Working Memory, by Ying Cai, Jacqueline M. Fulvio, Qing Yu, Andrew D. Sheldon, and Bradley R. Postle (2020).

Remembering recently encountered information requires holding in mind objects as well as their locations in the environment, commonly referred to as contextual binding (Yonelinas et al., 2019). For example, finding ingredients for a dinner recipe requires memory for both the object (e.g., potatoes) and its context, such as spatial location (e.g., bottom-left shelf in the kitchen).

Although the influence of context on long-term memory is well established (Robin, 2018), the influence of context on short-term memory is an emerging area of study. Recent models have proposed that spatial context is important for reducing interference between objects held in mind (Oberauer and Lin, 2017). Within this perspective, researchers have reinvestigated the neural basis of memory capacity, defined as the limited ability to hold information during the short term. One region, the intraparietal sulcus, was previously thought to be important for memory capacity because the delay-period activity in this region scales with the number of studied objects (Todd and Marois, 2004). However, in a subsequent experiment which indirectly tested memory for objects and context, the activity in the intraparietal sulcus was associated with context rather than memory capacity per se (Gosseries et al., 2018).

In the present study, Cai et al. (2020) directly examined object and spatial context representations in visual short-term memory. Using a clever behavioral design (Fig. 1), participants studied one oriented line (1O condition), three oriented lines (3O condition), or a combination of an oriented line, color, and luminance patch (1O1C1L condition). To-be-remembered items were displayed in different spatial locations at study. During test, the spatial location and category of the target was cued using a delayed recall task. Critically, the 3O condition required participants to remember both object and spatial context information, because items were sampled from the same category (i.e., category information was not sufficient to correctly retrieve the cued item from among the three held in memory). In contrast, the 1O1C1L condition did not require participants to remember spatial context, because items were sampled from different categories (i.e., category information was sufficient to correctly retrieve the cued item from among the three held in memory). This design elegantly manipulated the contextual binding demand between the 3O and 1O1C1L conditions, while matching the memory capacity demand (i.e., the 3O and 1O1C1L conditions both included three to-be-remembered items).

**Figure 1.**
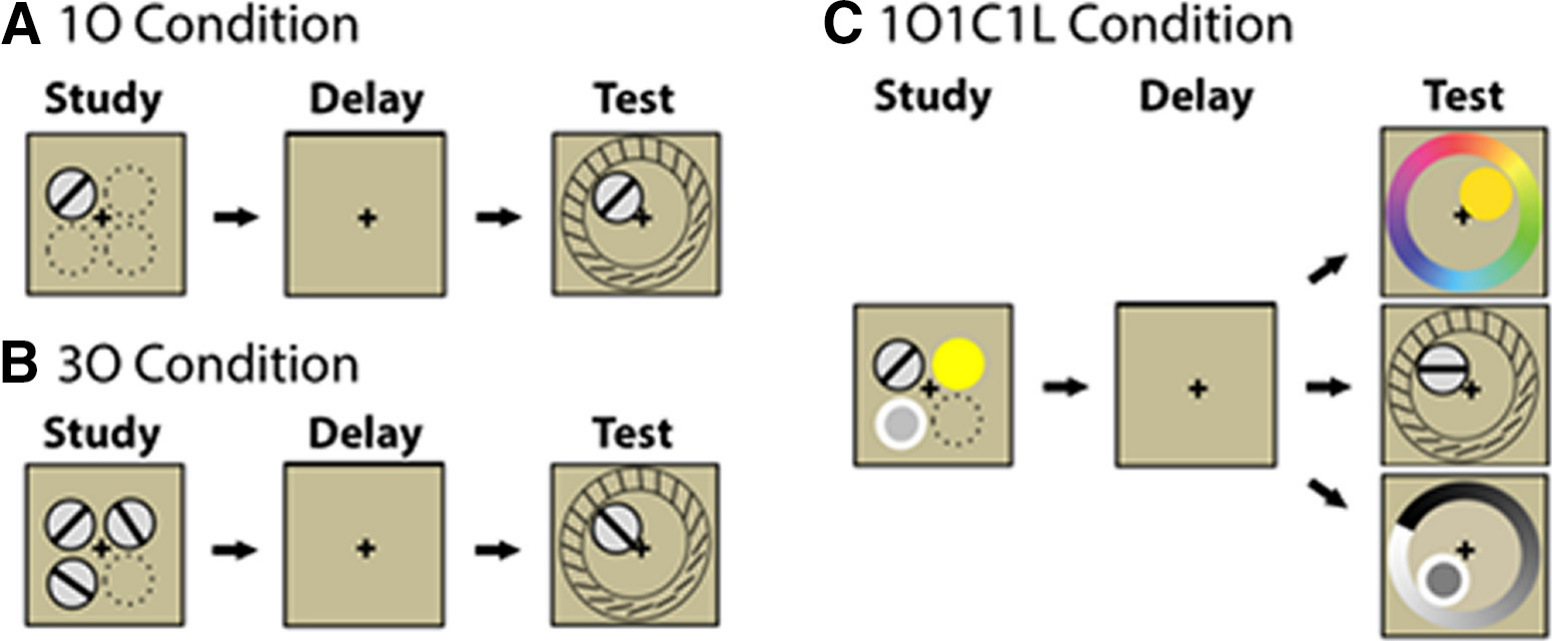
***A***, In the 1O condition, a single oriented line was displayed at study. ***B***, In the 3O condition, three oriented lines were displayed from the same category at study. ***C***, In the 1O1C1L condition, an oriented line, color, and luminance patch was displayed at study. In all conditions, the spatial location and category of a target item was cued at test and participants reconstructed the target on a circular stimulus space. Critically, only the 3O condition required participants to remember both object and spatial context information at study, because spatial location is required to disambiguate items from the same category at test. In contrast, the 1O1C1L condition did not require participants to remember spatial context at study, because the category is sufficient to disambiguate individual items at test. Figure adapted from Cai et al. (2020).

In a first experiment, the researchers examined whether regions including the intraparietal sulcus were sensitive to memory capacity. If a region responded only to memory capacity, then the 3O and 1O1C1L conditions (three studied items) should have greater activity then the 1O condition (one studied item). However, if a region were also sensitive to spatial context, then there should be a difference between the 3O and 1O1C1L conditions. This is because the additional task demand of contextual binding is needed to disambiguate objects in the 3O condition but not in the 1O1C1L condition. Contextual binding is not needed in the 1O1C1L condition because the stimulus category is cued at test, such that a participant can remember the target item without needing to remember its spatial location.

In a second experiment with preregistered hypotheses, the researchers used a swap model and an inverted encoding model to characterize the neural correlates of object and spatial context. The swap model estimates the likelihood that a participant incorrectly binds a nontarget item with the spatial location of the target item, serving as a behavioral estimate of contextual binding error. The inverted encoding model reconstructs the memory precision of object and spatial context information directly from neuroimaging data.

Across two experiments, the intraparietal sulcus and frontal cortex were found to be sensitive to contextual binding but not to memory capacity per se. The intraparietal sulcus was more active to the 3O condition compared with the 1O1C1L condition, because the 3O condition had a greater contextual binding demand (i.e., spatial location is needed to disambiguate multiple objects within the same category). Furthermore, spatial context directly influenced visual short-term memory: participants with fewer contextual binding errors (as measured by the swap model) had more precise object and spatial location representations in the occipital cortex and intraparietal sulcus (as measured by the inverted encoding model).

This study makes an important advance, as it is one of the first to investigate the neural basis of contextual binding in visual short-term memory. An intriguing area of future investigation may be whether these findings tested using simpler laboratory stimuli extend to complex naturalistic stimuli. Researchers studying episodic memory typically find regions in the medial temporal lobe rather than the intraparietal sulcus to be associated with spatial context (Yonelinas et al., 2019). One potential explanation for the difference between literatures may be related to the complexity of the stimuli (Cowell et al., 2019), as experiments in visual short-term memory typically favor well-controlled laboratory stimuli, whereas experiments in long-term memory typically favor complex naturalistic stimuli. For this reason, exploring the relationship between stimulus complexity and contextual binding may offer new insights into the similarities and differences between memory at different timescales.

Taken together, Cai et al. (2020) provide a compelling demonstration of the interplay between object and spatial context using neuroimaging, providing direct empirical support for contextual binding in visual short-term memory.

## Synthesis

Reviewing Editor: Christophe Bernard, INSERM & Institut de Neurosciences des Systèmes
